# Supplementary material for: Complexome profiling of the Chlamydomonas psb28 mutant reveals TEF5 as an early PSII assembly factor
Source: Plant Cell. 2025 Mar 17;37(6):koaf055. doi: 10.1093/plcell/koaf055 (PMC12164590; doi:10.1093/plcell/koaf055)
Supplement: koaf055_Supplementary_Data [file koaf055_supplementary_data.zip › psb28_interactive.zip!/plots/intensity/qualityPlots/peptides/CGL1_Cre03.g190150.t1.1.html]

### Cre03.g190150.t1.1

Trivial name: CGL1  
SeqCoverage [median]: 7.70  
Mass: 28.39 kDa  
  
 

| Sequence | MissedCleavage | Mass [Da] | wtIntensity | muIntensity | Score |
| --- | --- | --- | --- | --- | --- |
| ITGELEVSTAVR | 0 | 1273.69 | 758.41 | 1760.96 | 104.22 |
| YADVPTQR | 0 | 948.47 | 4291.02 | 5089.67 | 102.72 |
|  |  |  | 5049.4 | 6850.6 |  |
